# Rational Designing Microenvironment of Gas‐Diffusion Electrodes via Microgel‐Augmented CO_2_ Availability for High‐Rate and Selective CO_2_ Electroreduction to Ethylene

**DOI:** 10.1002/advs.202402964

**Published:** 2024-08-29

**Authors:** Hesamoddin Rabiee, Mengran Li, Penghui Yan, Yuming Wu, Xueqin Zhang, Fatereh Dorosti, Xi Zhang, Beibei Ma, Shihu Hu, Hao Wang, Zhonghua Zhu, Lei Ge

**Affiliations:** ^1^ School of Chemical Engineering The University of Queensland Brisbane QLD 4072 Australia; ^2^ Centre for Future Materials University of Southern Queensland Springfield QLD 4300 Australia; ^3^ Department of Chemical Engineering The University of Melbourne Melbourne VIC 3052 Australia; ^4^ School of Engineering Macquarie University Sydney NSW 2109 Australia; ^5^ Australian Centre for Water and Environmental Biotechnology (ACWEB) The University of Queensland St. Lucia QLD 4072 Australia; ^6^ School of Engineering University of Southern Queensland Springfield QLD 4300 Australia

**Keywords:** CO_2_ micro‐reservoir, electrochemical CO_2_ reduction reaction, gas‐diffusion electrode, GDE Microenvironment, Pyridine Microgels

## Abstract

Efficient electrochemical CO_2_ reduction reaction (CO_2_RR) requires advanced gas‐diffusion electrodes (GDEs) with tunned microenvironment to overcome low CO_2_ availability in the vicinity of catalyst layer. Herein, for the first time, pyridine‐containing microgels‐augmented CO_2_ availability is presented in Cu_2_O‐based GDE for high‐rate CO_2_ reduction to ethylene, owing to the presence of CO_2_‐phil microgels with amine moieties. Microgels as three‐dimensional polymer networks act as CO_2_ micro‐reservoirs to engineer the GDE microenvironment and boost local CO_2_ availability. The superior ethylene production performance of the GDE modified by 4‐vinyl pyridine microgels, as compared with the GDE with diethylaminoethyl methacrylate microgels, indicates the bifunctional effect of pyridine‐based microgels to enhance CO_2_ availability, and electrocatalytic CO_2_ reduction. While the Faradaic efficiency (FE) of ethylene without microgels was capped at 43% at 300 mA cm^−2^, GDE with the pyridine microgels showed 56% FE of ethylene at 700 mA cm^−2^. A similar trend was observed in zero‐gap design, and GDEs showed 58% FE of ethylene at −4.0 cell voltage (>350 mA cm^−2^ current density), resulting in over 2‐fold improvement in ethylene production. This study showcases the use of CO_2_‐phil microgels for a higher rate of CO_2_RR‐to‐C_2+_, opening an avenue for several other microgels for more selective and efficient CO_2_ electrolysis.

## Graphical abstract

The microenvironment of the gas‐diffusion electrodes is tuned by incorporating CO_2_‐phil microgels with amine moieties. Microgels as 3D polymer networks with tuneable chemical/physical properties can act as CO_2_ micro‐reservoirs to engineer the GDE microenvironment and boost CO_2_RR performance. Pyridine‐based microgels show bifunctional effects in enhancing CO_2_ availability through physical/chemical CO_2_ affinity and electrocatalytic CO_2_ reduction through their heterogeneous catalytic activity.

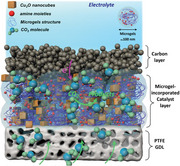

## 1 Introduction

Carbon dioxide (CO_2_) is commonly known as a greenhouse gas with adverse impacts on the environment and substantially contributes to global warming.^[^ ^1^ ^]^ On the bright side, once captured, CO_2_ can be an abundant carbon source that produces a variety of fuels and chemicals ranging from hydrocarbons to oxygenates.^[^ ^2^ ^]^ Electrochemical CO_2_ reduction reaction (CO_2_RR) is a promising technology powered by renewable or low‐carbon electricity to convert CO_2_ into value‐added chemicals.^[^ ^3^ ^]^ During the last two decades, much attention has been given to CO_2_RR, from developing advanced electrocatalysts to electrode/electrolyzer design and electrolyte effects.^[^ ^4^ ^]^ This has enriched our knowledge of CO_2_RR, and some start‐ups are already upscaling CO_2_ electrolyzers for simple products such as CO and formic acid. The low solubility of CO_2_ in aqueous solutions leads to insufficient availability of CO_2_ for conversion when CO_2_ is dissolved in the electrolyte. Therefore, this has urged researchers to turn their attention from the liquid‐phase reaction to the gas‐phase, where CO_2_ is continuously delivered to the system without dissolving in the electrolyte, where the reaction relies on limited CO_2_ transport from the bulk electrolyte.^[^ ^5^ ^]^

Gas‐diffusion electrodes (GDEs) have been developed to feed CO_2_ directly into the catalyst layer, shorten the CO_2_ transport pathway, and maximize local CO_2_ concentration in the vicinity of the catalyst in a flow‐cell electrolyzer.^[^ ^6^ ^]^ Using GDEs, mass transport limitation is alleviated, facilitating high‐rate CO_2_ electrolysis; therefore, ultrahigh current densities (>1 A cm^−2^) can be achieved, while conventional H‐cell reactors are typically limited to current densities of <60 mA cm^−2^. GDEs in the planar (or conventional),^[^ ^7^ ^]^ and recently, microtubular shapes,^[^ ^8^ ^]^ have been developed for CO_2_RR. Planar GDEs usually consist of a carbon‐based gas‐diffusion layer (GDL)^[^ ^6a^ ^]^ or PTFE‐based GDL^[^ ^9^ ^]^ coated with a catalyst layer. Electrolyte‐free, or membrane electrode assembly (MEA‐type) electrolyzers have also been developed to minimize the ohmic loss by stacking cathode GDE, ion‐exchange membrane, and anode together.^[^ ^10^ ^]^

Tuning GDEs’ microenvironment is of great importance to enhance CO_2_RR performance.^[^ ^11^ ^]^ The GDEs must be stable against flooding with minimal transport resistance for CO_2_ delivery and well‐constructed triple‐phase interfaces^[^ ^12^ ^]^ or liquid‐solid boundaries^[^ ^13^ ^]^ close to the catalytic active sites. The local microenvironment of GDEs plays a critical role in determining the mass transport and activity of gas‐involving electrocatalysis for stable and high‐rate CO_2_RR. Carbon‐based GDEs often suffer from flooding (i.e., ingress of the electrolyte into the inner layers and subsequently salt precipitation), causing performance degradation and parasitic hydrogen evolution reaction.^[^ ^14^ ^]^ To deal with this issue and control the diffusion of the electrolyte into the catalyst layer, remedies such as having a hydrophobic microenvironment by the addition of a hydrophobic agent^[^ ^15^ ^]^ or creating a dual layer of hydrophobic‐hydrophilic layers have been attempted in the literature.^[^ ^8a^ ^]^ Hydrophobic polytetrafluoroethylene (PTFE)‐based GDL as the substrate for GDEs has been recently introduced and optimized to replace carbon‐based GDLs.^[^ ^16^ ^]^ Although PTFE GDEs suffer from in‐plane resistance due to the non‐conductive nature of polymer substrates, recently novel current collectors have been designed to enable the scale‐up.^[^ ^16^, ^17^ ^]^

Moreover, it has been shown that increasing the local availability of the gas reactant is required for efficient electrolysis; for instance, by increasing CO_2_ availability by including CO_2_‐phil porous materials in the catalyst layer, one can increase the CO_2_ conversion efficiency to C_2+_ products.^[^ ^18^ ^]^ Nam et al. used a metal‐organic‐framework (MOF) layer under the catalyst layer to increase CO_2_ availability during CO_2_ electrolysis to ethylene at high current densities and observed that the MOF layer results in a FE of ethylene ∼50% at current densities over 800 mA cm^−2^.^[^ ^19^ ^]^ Another critical parameter is maximizing the triple‐phase interfaces (CO_2_‐electrolyte‐solid interface) and solid‐electrolyte interfaces, which are known as highly active catalytic areas for gas‐phase reactions. Multiple studies have attempted to add rigid colloids such as PTFE or SiO_2_ to the catalyst layer for this purpose by creating a semi‐hydrophobic microenvironment in the catalyst layer.^[^ ^15^, ^20^ ^]^ The local CO_2_ concentration is significant for the formation of C_2+_ products where critical C─C coupling is necessary for the efficient formation of products such as ethylene.^[^ ^21^ ^]^ Through engineering the GDE microenvironment for high‐performance CO_2_RR and maximum CO_2_ consumption and applying it to PTFE‐based GDEs, high‐rate and stable ethylene production could be achieved.

A great candidate for tuning the microenvironment in GDEs could be CO_2_‐phil microgels with amine moieties in their structure. Microgels are 3D crosslinked polymer networks in a roughly spherical shape, synthesized via emulsion polymerization, and their unique properties have been investigated for various applications such as gas adsorption,^[^ ^22^ ^]^ membranes,^[^ ^23^ ^]^ osmosis,^[^ ^24^ ^]^ and drug delivery.^[^ ^25^ ^]^ Microgels are also highly tunable, and their physical/chemical, and mechanical properties can be functionalized based on the target application.^[^ ^26^ ^]^ However, their application for electrocatalysis has yet to be studied, as they are distinctly different from common colloids^[^ ^27^ ^]^ that have been added to the catalyst layer of GDE to tune the microenvironment in CO_2_RR.^[^ ^15^, ^20^ ^]^ The highly tunable physical/chemical properties of microgels,^[^ ^26^ ^]^ CO_2_ storage capability,^[^ ^28^ ^]^ and 3D structure to create well‐constructed triple‐phase interfaces in GDEs, compared with commonly used colloids, offer opportunities to increase the CO_2_ local concentration to enhance reaction rate in the CO_2_RR.

Herein, given the unique and tunable properties of microgels, we explore their function to tune the microenvironment of PTFE‐based GDEs for CO_2_RR to ethylene through rational design of CO_2_‐phil microgels with pyridine‐based amine moieties. The microgels with the heterocyclic amine backbones in their structure act as CO_2_ micro‐reservoirs in the catalyst layer and improve CO_2_ availability. In the meantime, the microgels prepared with pyridine groups show dual functionality, not only providing CO_2_ availability to achieve stable selectivity at high current density but also leading to enhanced FE of ethylene as compared with the pristine GDE and GDE with pyridine‐free microgels. It was noted that balancing the CO_2_ availability and catalytic activity through the crosslinking ratio of the microgels resulted in flexing the intrinsic advantages of microgels over colloids owing to their 3D structure. The GDE with 20wt.% PVP microgels showed superior CO_2_RR‐to‐ethylene performance, and high FE of ethylene (56%) was achieved at a significantly higher current density (up to 700 mA cm^−2^) as compared with the GDE without microgels whose lower ethylene FE was capped at 300 mA cm^−2^. The results of the MEA cell also showed a similar trend, and the GDE with microgels‐augmented CO_2_ availability showed over a twofold increase in the partial current density of ethylene compared to the GDEs without microgels. This study showcases the potential of using functionalized and 3D polymer particles to boost CO_2_RR performance for C_2+_ products, and it can open an avenue toward their future applications for CO_2_RR and other electrocatalytic reactions in which optimizing the electrode microenvironment matters.

## 2 Results and Discussion

### 2.1 Synthesis and Characterizations

**Figure 1:**
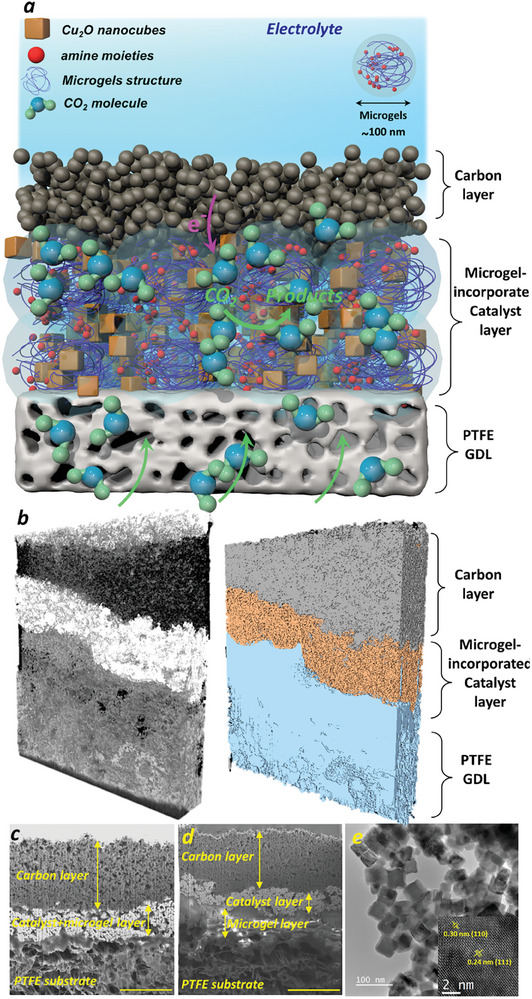
a) Schematic of a GDE consisting of a catalyst layer, modified with microgels, and a carbon black top‐layer on PTFE substrate; b) 3D constructed structure of the GDE with 20 wt.% PVP microgels incorporated into the catalyst layer; cross‐sectional FIB‐SEM image of c) the GDE showing the carbon layer on top, catalyst layer with 20 wt.% PVP microgels in the middle, and PTFE substrate below; d) the GDE with a layer of PVP microgel sandwiched in between the catalyst layer and PTFE substrate (scale bar: 5 µm); e) TEM and high‐resolution TEM image of Cu_2_O nanocubes used as the catalyst to fabricate GDE.

We studied the role of CO_2_‐phil microgels in increasing the CO_2_ availability in the vicinity of the catalyst layer. Catholyte and anolyte solutions were circulated in different sections, and CO_2_ was fed to the electrolyzer from the GDE's back (Figure S3, Supporting Information). The schematic of a GDE, the position of different layers, and the CO_2_ delivery path can be found in **Figure** **1**a. The 3D‐constructed FIB‐SEM of the GDE also showed the existence of three distinctive layers (Figure 1b). In addition to the incorporation of microgels in the catalyst layer via mixing with catalysts (Figure 1a), the microgels were also sandwiched between the PTFE GDL and catalyst layer (Figure S4, Supporting Information). The layers of the GDE (PTFE layer, catalyst layer, and carbon layer) were seen in FIB‐SEM images (Figure 1c,d) for both cases, indicating the successful fabrication of the GDEs. The PTFE layer provides a robust and flooding‐resistant substrate and helps to uniformly deliver CO_2_ to the catalyst layer while the carbon top layer acts as the current collector. The high‐resolution transmission electron microscopy (HRTEM) images of the Cu_2_O catalyst (Figure 1e; Figure S6, Supporting Information) presented a uniform cubic shape with the size of ≈20 nm and lattice sizes of 0.3, 0.24, and 0.21 nm attributed to crystal facet of (100), (111) and (200), respectively, consistent with the XRD spectra of the catalyst powders (Figure S7, Supporting Information). It should be noted that cubic shape has shown higher selectivity toward C_2+_ products than other copper‐based catalysts,^[^ ^29^ ^]^ and that is why it is selected as a simple catalyst to showcase the microgels‐incorporated GDEs in this study. Uniform distribution of Cu and O atoms was also observed in the EDAX mapping of HRTEM images (Figure S8, Supporting Information). The XPS spectra of the catalyst powders were recorded and showed the oxidation of the catalyst with characteristic peaks related to Cu/Cu^+^, and weak satellites attributable to Cu^2+^ (Figure S9, Supporting Information).

**Figure 2:**
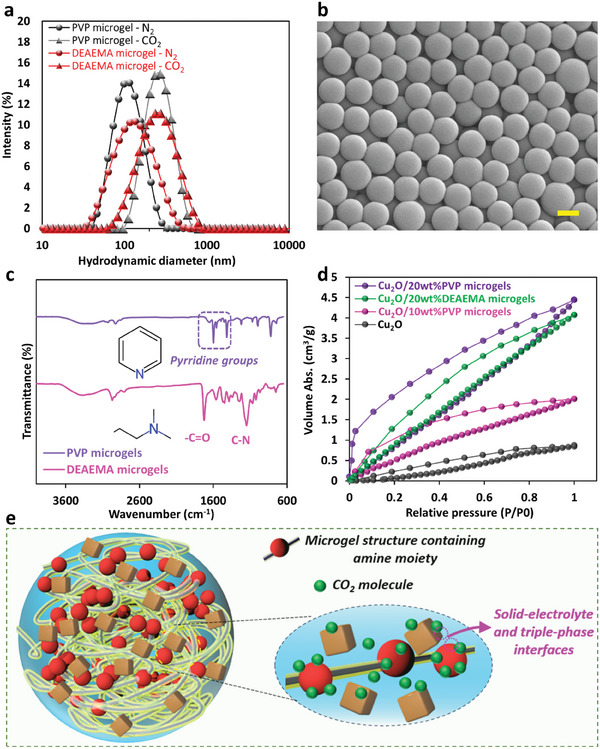
a) Hydrodynamic diameter distribution of PVP and DEAEMA microgels in CO_2_‐ and N_2_‐saturated solution; b) SEM of PVP microgels (scale bar: 100 µm); c) FTIR spectra of PVP and DEAEMA microgels; d) CO_2_ adsorption−desorption isotherm curves of Cu_2_O nanocubes and Cu_2_O nanocubes with 20 wt.% of PVP microgels; e) Schematic illustration of the formation of triple‐phase boundaries and solid‐electrolyte interface in the microgels 3D structure and proximity of the catalyst, the interfaces of amine‐containing microgels (shown in highlight yellow) act as CO_2_ reservoirs for sufficient CO_2_ availability.

The hydrodynamic diameter of microgels was measured using dynamic light scattering (DLS). It was found that both PVP and DEAEMA microgels are roughly 100 nm (**Figure** **2**a), consistent with the SEM results (Figure 2b). The dilute suspension of the microgels was tested in DLS saturated with either N_2_ or CO_2_. It was noticed that the diameter of the microgels increases in the CO_2_‐saturated solution. It was seen that the CO_2_‐saturated solution became transparent after CO_2_ saturation, while it was milky when saturated with N_2_ (Figure S10, Supporting Information). The microgels containing tertiary amine moieties are reported to be CO_2_‐responsive; therefore in the presence of CO_2_, they become protonated and swell, depending on the pK_a_ of the microgels and the pH of the environment.^[^ ^24^, ^30^ ^]^ It should be noted that although pyridine is not classified as a tertiary amine, it has chemical properties similar to tertiary amines,^[^ ^31^ ^]^ and its CO_2_‐responsive behavior through the protonation of amine moieties has been reported.^[^ ^32^ ^]^ The pKa for poly (DEAEMA) and poly (4‐vinyl pyridine) are reported to be ≈7.4 and 5.6, respectively.^[^ ^33^ ^]^ Moreover, as reported in several studies, the pH in the vicinity of the catalyst layer during the CO_2_ reduction reaction is alkaline.^[^ ^34^ ^]^ Therefore, the microgels are in the unswollen state during CO_2_RR, and the microgels‐incorporated catalyst layer will neither increase the water content of the catalyst layer nor lead to delamination of the catalyst layer due to swelling of microgels. The contact angle analysis of the GDE with 20 wt.% PVP microgels showed no significant increase in the wettability as compared with the GDE without microgels (Figure S11, Supporting Information), and GDEs showed a hydrophobic surface due to the hydrophobicity of Nafion binder with polytetrafluoroethylene backbone.^[^ ^35^ ^]^ The GDE also kept its surface hydrophobicity after CO_2_RR tests (Figure S11c, Supporting Information). Hydrophilic GDE surface will increase the accessibility of water in the catalyst layer, blocking CO_2_ transport pathways and disrupting the formation of triple‐phase interfaces, which is not ideal for a well‐constructed triple‐phase interface.^[^ ^36^ ^]^

FTIR spectra of PVP and DEAEMA microgels exhibit the formation of polymeric bonds and their characteristic peaks regarding the existence of amine groups in their structure. As can be seen in Figure 2c, the characteristic vibrations of the pyridine ring were present at 1598, 1556, 1461, and 1416 cm^−1^ for the PVP microgel, while the peaks at 1146 and 1724 cm^−1^ are respectively attributed to C─N and ‐C═O bands on DEAEMA microgels.^[^ ^37^ ^]^ The CO_2_ adsorption‐desorption isotherms of Cu_2_O and Cu_2_O/microgels mixture exhibit higher CO_2_ adsorption capacity of Cu_2_O/microgels, attributed to 1. Porous structure of microgels with capability for gas storage, and 2. the interaction between CO_2_ and microgels with amine‐containing polymeric branches, indicating the CO_2_‐philicity of microgels.^[^ ^38^ ^]^ As shown in Figure 2d, increasing microgel content in the mixture resulted in a gradual increase in CO_2_ uptake for 10 wt.% and 20 wt.% PVP microgels, and similarly for 20 wt.% DEAEMA microgels mixture with Cu_2_O. When incorporated into the catalyst layer, microgels with a polymeric network of amine moieties act as micro‐reservoirs to increase CO_2_ availability and supply sufficient CO_2_ in the catalyst layer.^[^ ^20^, ^39^ ^]^ The enhanced CO_2_ adsorption is likely associated with the reversible formation of an adduct by CO_2_ and the amine moieties of the microgels.^[^ ^40^ ^]^ In addition to the physisorption, attributed to the 3D and porous structure of microgels, CO_2_ temperature programmed desorption analysis exhibited a desorption peak at 80–100 °C (centered at 93 °C), indicating the chemical affinity of CO_2_ molecules on heterocyclic amine moieties of PVP microgels (Figure S12, Supporting Information). It should be noted that water/humidity increases CO_2_ capture by amine groups,^[^ ^41^ ^]^ and this will happen in the microenvironment of GDEs where the catalyst layer containing microgels becomes wet in contact with the electrolyte (or humidified CO_2_ in MEA cell). The schematic of the formation of triple‐phase boundaries and solid‐electrolyte interface in the catalyst layer incorporated with the amine‐containing microgels is presented in Figure 2e. As illustrated, the 3D structure of microgels provide additional interface formation for the Cu_2_O catalyst and pyridine (as co‐catalyst) and amine moieties act as CO_2_ micro‐reservoirs providing sufficient CO_2_ availability which is critical for efficient CO_2_‐to‐C_2+_ reaction at elevated current densities, as investigated later.

### 2.2 Electrocatalytic CO_2_RR Performance

We tested a heterocyclic amine‐containing microgel with a pyridine ring, poly 4‐vinyl pyridine (PVP) (Figure S1, Supporting Information), which is a CO_2_‐switchable polymer and has shown activity for CO_2_ reduction both in forms of homogenous and heterogenous catalysis.^[^ ^32^, ^42^ ^]^ In addition, microgels of poly(*N*, *N*‐(diethylamino)ethyl acrylamide (Poly‐DEAEMA or PDEAEMA), a well‐studied CO_2_‐responsive polymer with tertiary amines groups (Figure S1, Supporting Information) were synthesized to compare with PVP microgels performance.^[^ ^30^, ^43^ ^]^ The aim is to confirm and take advantage of the dual functionality of these microgels to enhance CO_2_RR via both catalytic interferences and also improved CO_2_ availability and triple‐phase formation. Moreover, the geometry of microgel incorporation (mixed with the catalyst or as a separate underlayer) and its degree of crosslinking are investigated. The studies on using amine‐functionalized GDEs to facilitate CO_2_RR have primarily focused on modifying the catalyst surface (mostly Cu/Ag/carbon foil as the cathode catalyst).^[^ ^44^ ^]^ Whereas, using the amine‐containing 3D network microgels in the vicinity of the catalyst has intrinsic advantages in creating triple‐phase interfaces, acting as CO_2_ micro‐reservoirs, and better processability for larger‐scale fabrication of GDEs due to the well‐established emulsion polymerization.

**Figure 3:**
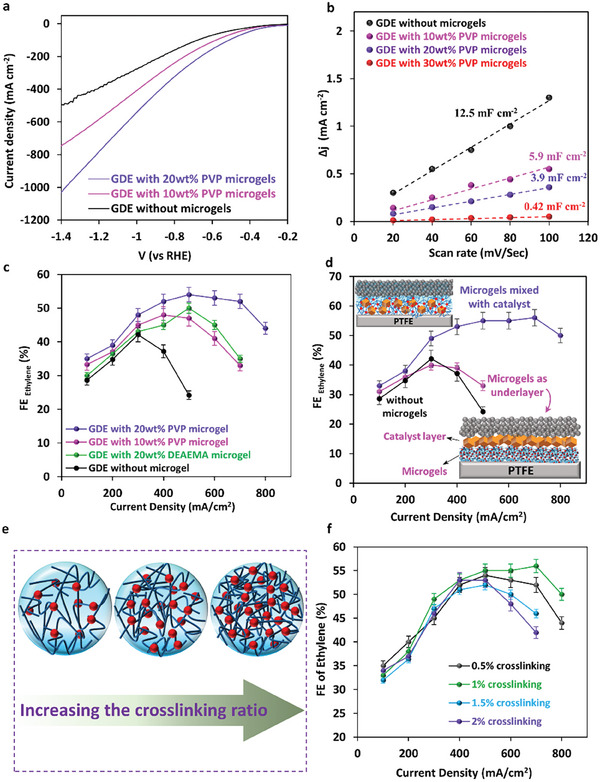
a) Linear sweep voltammetry of GDEs with and without PVP microgels; b) FE of ethylene for GDEs with and without PVP microgels; c) comparison in ethylene selectivity of GDEs with 20 wt.% PVP and DEAEMA microgels; d) effect of the geometry of microgels addition to the GDE on ethylene selectivity, in both cases 20 wt.% PVP microgels are incorporated to the GDE; e) schematic of microgels structure with increasing the crosslinking ratio; f) effect of microgels crosslinking ratio on ethylene selectivity.

PVP microgels were incorporated into the catalyst layer of the GDE and the LSV of the GDEs showed an increase in the current density after incorporation of microgels up to 20 wt.% (**Figure** **3**a). This is attributed to the increase in the triple‐phase formation in the catalyst layer of GDE and improved CO_2_ availability and enriched CO_2_‐electrolyte‐catalyst interface by adopting CO_2_‐phil microgels.^[^ ^45^ ^]^ A moderate wettability of the catalyst layer is required to establish triple‐phase interfaces. If the electrolyte soaks the catalyst layer, it will cause CO_2_ mass transport resistance, while a lack of electrolyte in the catalyst layer leads to poor catalyst‐electrolyte contact, hindering CO_2_RR activity.^[^ ^13^ ^]^ The electrochemical active area (ECSA) measurement was carried out (via CV cycles, Figure S13, Supporting Information) as a representative of the wetting area of the electrodes.^[^ ^20d^ ^]^ The results showed a decrease with the incorporation PVP microgels to the GDE, from 12.5 mF cm^−2^ for the GDE without microgels to 5.9 and 3.9 mF cm^−2^ for the GDE with 10 wt.% and 20 wt.% PVP microgels added to the catalyst layer, respectively (Figure 3b). For the GDE with 30 wt.% PVP microgels, it significantly dropped to 0.43 mF cm^−2^, indicating the disturbed formation of triple‐phase interfaces, and that having a moderate amount of microgels leads to the optimal local CO_2_/H_2_O ratio in the catalyst layer.^[^ ^46^ ^]^ As the polymeric microgels are non‐conductive, having 30 wt.% microgels content attributed to the increase of electron/charge transfer resistance in the catalyst layer, as an excessive voltage was observed for the GDE with 30 wt.% microgels. Moreover, higher microgel content might disrupt the CO_2_ delivery channels within the catalyst layer and adversely affect optimized triple‐phase formation,^[^ ^15^, ^20^ ^]^ therefore, up to 20 wt.% microgel incorporation was further tested in this study. The LSV of the GDE with 20 wt.% microgels in the Ar atmosphere was also obtained and showed a significant difference compared to when CO_2_ was used (Figure S14, Supporting Information). The observed current in the Ar atmosphere is related to H_2_ evolution, indicating the fabricated GDE's activity toward CO_2_ reduction reaction.

To investigate the selectivity, GDEs were tested in constant current density, and the FE of products was measured. Looking at the ethylene selectivity of the pristine GDE without microgels (Figure 2c), it was seen that FE of ethylene was capped at 43% at ≈300 mA cm^−2^ (−1.1 V vs. RHE), and further increasing the current density led to excessive hydrogen evolution reaction (Figure S15a, Supporting Information). Therefore, the partial current density of ethylene was capped at 148.5 mA cm^−2^ (at a total current density of 400 mA cm^−2^). The reduction of ethylene selectivity with increasing current density is attributed to the lack of CO_2_ availability in the vicinity of the catalyst layer for moderate‐to‐high current densities; therefore, high ethylene production could not be achieved. It should be noted that the carbon top layer also plays a role in the production of CO at lower potential ranges and generation of ^*^CO, which can be further reduced to ethylene as the applied current density increases.^[^ ^47^ ^]^ It has also been reported that having a carbon layer on top of the catalyst layer provides extra pathway channels for CO_2_ and can facilitate CO_2_ transport to the catalytic active sites.^[^ ^48^ ^]^ The PVP microgels‐incorporated GDEs showed a considerable increase in the ethylene partial current density. PVP microgels were added to the catalyst ink up to 20 wt.% relative to Cu_2_O, and CO_2_RR performance showed that FE of ethylene increased to up to 55–56% for the GDE with 20 wt.% relative content of PVP microgel. Therefore, stable production of ethylene was observed at current densities as high as 700 mA cm^2^ (−1.15 V vs. RHE). The stable FE of ethylene at higher current densities indicates improved CO_2_ availability, and therefore the catalyst layer performance is not deprived of CO_2_ feed.^[^ ^19^ ^]^

The increase in the FE of ethylene is also due to the catalytic effect of pyridine moieties in PVP microgels, making their incorporation with dual effect both as the CO_2_ micro‐reservoir and also as active co‐catalyst for CO_2_ reduction.^[^ ^49^ ^]^ The mechanism for ethylene production is through C─C coupling, a well‐known route for the formation of C_2+_ products during CO_2_RR on Cu catalysts.^[^ ^50^ ^]^ Herein, the microgels contribute to a higher ethylene selectivity supposedly through 1. Limiting proton diffusion into the catalyst layer as the existence of microgels in close vicinity of the catalysts and their unswollen state leads could tune the wettability and hinder the excessive proton diffusion into the catalyst layer, thus increasing the local pH and suppressing the H_2_ evolution reaction,^[^ ^40^, ^51^ ^]^ 2. The pyridine moieties of microgels are reported to enhance ^*^CO coverage and result in higher local CO partial pressure, and therefore leading to improved C_2+_ product formation through C─C coupling.^[^ ^52^ ^]^ The *operando* Raman spectroscopy study on the Cu catalyst modified by a pyridine film showed a higher intensity for the band attributed to C≡O stretching of the adsorbed ^*^CO for the pyridine‐modified electrode as compared with the pristine Cu.^[^ ^52a^ ^]^ Therefore, by establishing a ^*^CO‐rich microenvironment close to the Cu catalyst and increasing the retention time of the in situ generated CO near the catalyst, C─C coupling and C_2+_ product generation are improved.^[^ ^53^ ^]^

In addition, there have been reports on the CO_2_RR activity of protonated pyridines in reducing the activation energy of the CO_2_ reduction reaction and acting as a co‐catalyst in the vicinity of a metal catalyst.^[^ ^49^, ^54^ ^]^ Overall, physically reserving and chemically activating CO_2_ by microgels result in facilitated reaction kinetic of the microgel‐incorporated GDEs.

Looking at the effect of the microgels loading on the CO_2_RR performance of the GDEs, incorporating 10 wt.% microgels improved ethylene production and increased the capped ethylene partial current density to 400 mA cm^−2^ (−1.05 V vs. RHE). PVP microgels improved both FE and current density of the GDE as compared with the GDE without microgels, and this improvement continues further for the one with 20 wt.% PVP microgels (Figure 3c). Therefore, microgels not only boost CO_2_ availability for a higher ethylene selectivity but also due to their 3D structure they create active pathways within the catalyst layer and lead to the optimal local CO_2_/H_2_O ratio. This results in higher chances of triple phase interface formation, and consequently, higher current densities were observed when the microgels content increased to up to 20 wt.%.

To further confirm the improvement in the incorporation of PVP microgels is related to their dual functionality with both capture and reduction properties, PDEAEMA (poly‐DEAEMA) microgels were considered as an alternative to PVP microgels. PDEAEMA contains tertiary amines and is a CO_2_‐switchable polymer,^[^ ^55^ ^]^ but unlike heterocyclic cyclic amines such as pyridine, pyridazine, pyrazole, or imidazole,^[^ ^42^, ^49^, ^56^ ^]^ CO_2_ reduction activity has not been reported for them. PDEAEMA was added to the catalyst ink at 20 wt.%, and GDEs were tested for CO_2_RR performance. As compared with the GDEs without microgels, an increase in both FE of ethylene and current density was seen for the GDE with 20 wt.% PDEAEMA microgel and FE of ethylene just over 50% at 500 mA cm^−2^ current density was achieved (Figure 3c), indicating the effect of amine moieties of DEAEMA microgel as CO_2_ reservoir for the catalyst layer. However, compared with 20 wt.% PVP microgels, ethylene partial current density is less than that observed for PVP microgels, which indicates the catalytic role of pyridine moieties of the microgels and better performance of PVP microgels to improve GDEs for CO_2_RR through dual functionality effect.

### 2.3 Geometry of GDE and Microgels

To get a better insight into the co‐catalytic effect and creation of triple‐phase interfaces in the catalyst layer after microgels incorporation, we investigated the geometrical aspect of the microgel layer, and PVP microgels were deposited as a sub‐layer under the catalyst layer (Figure 1c; Figure S4, Supporting Information) as compared to mixing with the catalyst in one layer (Figure 1b). In this case microgels were not mixed with the catalyst and it was thought that a microgel layer could act as a high‐concentration CO_2_ layer just under the catalyst layer (0.4–0.45 mg cm^−2^ loading for the microgels layer). As compared with the GDEs prepared by adding the microgels to the catalyst ink, this arrangement showed less FE of ethylene at the high current densities, and the GDE with the sub‐layer showed similar performance compared to the GDE without microgels (Figure 3d), indicating that the sub‐layer is not performing well to improve the CO_2_ availability. However, when the microgels are incorporated in the close vicinity of the catalyst (mixed in ink), they are more likely to uniformly provide sufficient CO_2_ for the catalyst during CO_2_RR, whereas in the separate layer configuration, there will be extra mass transport for CO_2_, and microgels cannot establish triple‐phase interfaces in the vicinity of catalysts. Moreover, having microgels in the proximity of the Cu_2_O, and carbon top‐layer where charge/electron transfer occurs provides the right environment for them to act as a co‐catalyst, whereas, in the sublayer scenario (Figure 1c; Figure S4, Supporting Information), microgels are less likely to expose their electrocatalytic effect. These results suggest that having 3D‐structured microgels in the proximity of Cu_2_O is critical to ensure both local CO_2_ availability and the co‐catalytic advantages of PVP microgels.

We then studied the structure of microgels and their impact on the CO_2_RR performance of GDE. Microgels are cross‐linked 3D polymer networks; therefore, the degree of crosslinking can affect their properties.^[^ ^57^ ^]^ The PVP microgels were initially synthesized with 0.5 wt.% crosslinker as the minimum required amount, as we observed that having less than 0.5 wt.% crosslinking did not result in successful polymerization and synthesis of the microgels. Further, PVP microgels with 1, 1.5, and 2 wt.% crosslinking ratios were synthesized to investigate the microgel structure effect on CO_2_RR. Increasing the crosslinking ratio did not result in a noticeable increase in the diameter of microgels (Figure S16, Supporting Information), meaning that microgels became denser. It was observed that increasing the concentration of crosslinking agent to 1 wt.% led to a better activity as compared with 0.5 wt.% crosslinking and achieving a higher FE of ethylene at 700mA cm^−2^ (Figure 3f; Figure S17, Supporting Information), while for the GDE prepared with PVP microgels from 0.5 wt.% crosslinking, FE of ethylene slightly decreased after 600 mAcm^−2^. This shows that the microgels with 0.5 wt.% had less structural density which act as CO_2_ storage sites. Increasing the crosslinking concentration in microgels will result in higher amine moieties however higher crosslinking increases the density^[^ ^57^ ^]^ (schematically shown in Figure 3e). Interestingly, further increase in crosslinking ratio to 1.5 and 2 wt.% did not favor better CO_2_RR performance, as seen in Figure 3f. The optimal crosslinking in the microgels can affect CO_2_RR performance as having looser microgel structure results in better mass transport of CO_2_ and intermediates in the microgels, and easier accessibility of the CO_2_ to the proximate catalysts. In addition, microgels with balanced amine moieties content and mass transfer resistance can create well‐established triple‐phase interfaces (solid‐gas‐electrolyte), resulting in better electrocatalytic performance.^[^ ^12^, ^13^ ^]^ Microgels with 1.5 and 2 wt.% crosslinking agent have higher solid (polymer) content and, therefore, less space for creating triple‐phase interfaces within their structure. These results indicate that the 3D structure of the microgels is important in providing spaces for triple‐phase interfaces and CO_2_ storage and consequently can affect the CO_2_RR performance of GDEs. The ethylene partial current density of 392 mA cm^−2^ (−1.1 V vs. RHE) achieved for the GDE with 20 wt.% PVP microgels and 1 wt.% crosslinking ratio exhibited a nearly twofold increase in partial current density of C_2_H_4_ compared to the GDE without microgels, indicating the merit of improved local CO_2_ concertation for facilitating high‐rate CO_2_RR.

### 2.4 Insights into Microenvironment

To get further mechanistic insights into the solid–liquid–gas interface microenvironment of the microgel‐modified GDEs and understand CO_2_ mass transport in the catalyst layer, the Nernst diffusion process was tested by electrochemical impedance spectroscopy (EIS), which can be represented in circuit modeling as equivalent impedance *Z*_d_. The Nernst diffusion layer is defined as a virtual layer of CO_2_ concentration gradient interval from the electrode surface to where the concentration of CO_2_ reaches the bulk concentration.^[^ ^58^ ^]^ The thickness of the diffusion layer (*δ*) and limiting current density (*J_lim_*) for CO_2_RR are correlated as follows: *J_lim_ = nFD_0_C_0_/δ*, where *D_0_* and *C_0_* are the diffusion coefficient and solubility of CO_2_ in the electrolyte, respectively, n is the number of electron transfer in the reaction and F is the Faraday constant. Based on the above equation, reducing the thickness of the diffusion layer leads to effectively a higher limiting current density for CO_2_RR. The thickness of the diffusion layer (*δ*) can be determined via $\delta \;=\sqrt{3R_{d}D_{0}C_{d}}$, where *R*_d_ and *C*_d_ are the equivalent resistance and capacitance of the diffusion layer describing the ability of conducting and storing electric charge of this layer, respectively. *D_0_* was calculated via a thermodynamic equation at the value of 0.1578.^[^ ^46^ ^]^

**Figure 4:**
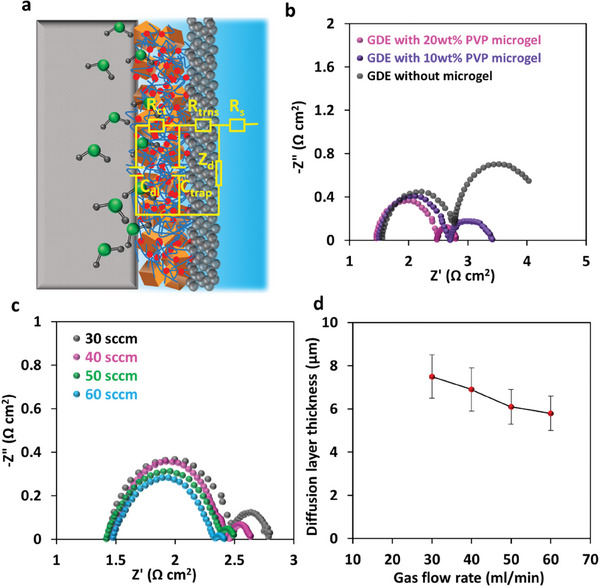
a) A proposed circuit model for the electrodes, R_s_ is the electrolyte resistance, R_ct_ and C_trap_ are electron transport resistance and trap capacitance of carbon top layer, R_ct_ and C_dl_ are charge transfer resistance and double layer capacitance in the catalyst layer, Zd is the Nernst diffusion impedance; b) EIS spectra acquired for different GDEs (−1.0 V vs. RHE, 30 ml min^−1^ CO_2_ gas flow); c) EIS spectra acquired for the GDE with 20 wt.% PVP microgels at −1.0 V vs. RHE under various CO_2_ gas flow rates; d) Diffusion layer thicknesses derived from the EIS spectra in (c).

By acquiring EIS spectra for GDEs under CO_2_RR and fitting the spectra with the circuit model in **Figure** **4**a, (where R_ct_ is equal to R_d_, C_dl_ is equal to C_d_) using EIS Spectrum Analyser, R_ct_ and C_dl_ are obtained (Table S1, Supporting Information) and the diffusion layer thicknesses can be calculated.^[^ ^46^, ^59^ ^]^ Z_d_ can be obtained via Z_d_ = R_d_ – jω $R_{d}^{2}$ C_d_, where J is the imaginary unit, and ω is the angular frequency. From Figure 4b, it can be seen that the GDE without microgels showed a larger diffusion impedance at low‐frequency region, and the calculated diffusion layer thickness decreased from 15.3 ± 0.8 µm for the GDE without microgels to 7.3 ± 0.3 µm for the GDE with 20 wt.% PVP microgels. This indicates the improvement in CO_2_ availability and mass transport and reduced diffusion layer thickness provided by the incorporation of the microgels into the catalyst layer of GDE.^[^ ^15b^ ^]^ Moreover, the reduction in the diffusion layer thickness means more chances for the formation of a triple‐phase interface and a higher limiting current density (via *J_lim_ = nFD_0_C_0_/δ*), consistent with the current density results observed after microgels incorporation. Further, the effect of CO_2_ flow rate was studied and EIS spectra for GDEs under CO_2_RR with different CO_2_ flow rates were recorded (Figure 4c). A relatively linear relationship between the CO_2_ flow rate and the diffusion layer thickness was observed (Figure 4d), consistent with other similar studies where particles were added to the catalyst layer of a GDE to improve CO_2_ mass transport and microenvironment for CO_2_RR.^[^ ^15^, ^20^ ^]^ This indicates that increasing the CO_2_ flow rate leads to a higher local pressure or local CO_2_ concentration, consequently improving CO_2_ mass transport and CO_2_ electrolysis performance.^[^ ^60^ ^]^

### 2.5 MEA Performance of Microgel‐Incorporated GDEs

**Figure 5:**
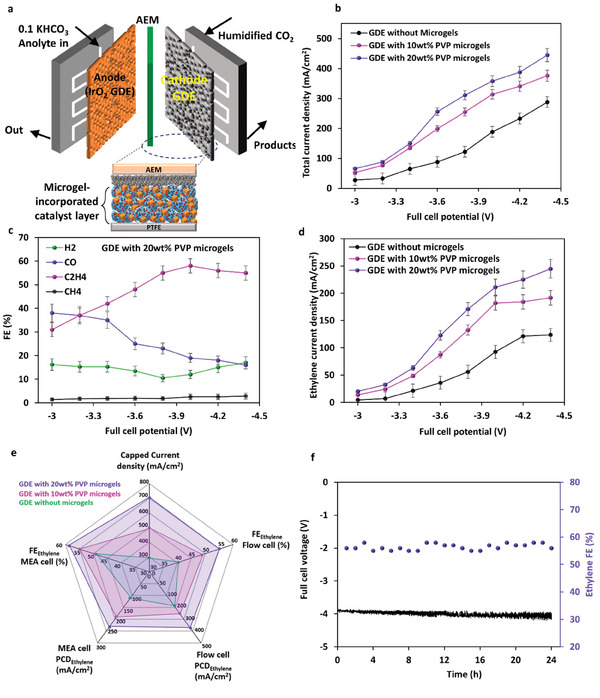
a) Schematic of MEA cell and GDE location in the cell to evaluate microgels‐incorporated GDEs in a zero‐gap electrolyzer; b) total current density of MEA cell as a function of full cell voltage for the GDEs with and without microgels; c) Product selectivity of the GDE with 20wt.% microgels as a function of full cell voltage; d) the partial current density of ethylene for the GDE with and without microgels; e) Spider web performance of GDEs with and without microgels for their key results in alkaline and MEA cells; f) Stability of MEA cell for 24 h test at 350 mA cm^−2^ and the measured FE of ethylene.

We further tested the performance of the GDEs in a catholyte‐less membrane‐electrode assembly (MEA) cell (**Figure** **5**a).^[^ ^10a^ ^]^ The MEA cell used an IrO_2_ GDE as the anode, which was pressed into the anion exchange membrane and cathode GDE. 0.1 M KHCO_3_ was used as the anolyte with humidified CO_2_ feeding into the reactor, operated at ambient pressure and temperature.^[^ ^61^ ^]^ The humidity of the supplied CO_2_ is necessary for ethylene production as this humidity, as well as the water transport from the anolyte, has been reported to provide the protons needed for ethylene production, in addition to enhancing the water activity and microenvironment, and consequently CO_2_RR performance.^[^ ^62^ ^]^ The GDE with and without PVP microgels were tested in MEA configuration at constant full‐cell voltages, and partial current densities for each product were calculated and compared.

Looking at the performance of the GDEs with and without microgels, there was an increase in the overall current density after the incorporation of PVP microgels within the tested voltage range (Figure 5b). This is consistent with the trend observed for the alkaline flow cell, indicating the effect of microgels to improve CO_2_RR in both systems. For the GDE with 20 wt.% of PVP microgels, the FE of ethylene reached over 50% from a cell voltage of −3.8 V, increasing to 58% at −4 V (current density of 358 mA cm^−2^) with slightly decreasing at higher voltages, along with an increase in FE of H_2_ (Figure 5c). For the GDE without any microgels, the highest FE of ethylene (50%) was achieved at a higher voltage (−4.2 V) (Figure S18, Supporting Information), showing that microgels led to achieving higher ethylene selectivity at a lower voltage. The combination of higher FE of ethylene and toral current density for the GDE with 20 wt.% PVP microgels led to an over twofold higher partial current density of ethylene over the GDE without microgels, indicating the effectiveness of this approach for ethylene production (Figure 5d). Similarly, the highest partial current density of CO production was seen for the GDE with PVP microgels (Figure S18, Supporting Information), which could be attributed to the promoted ^*^CO dimerization at high current densities. It can be concluded that the principle behind microgel‐augmented GDEs was similar to what was observed for the flow cell via improving CO_2_ availability in the catalyst layer and near the catalytic active sites and improving C_2_H_4_ production.

Analysis of liquid samples was also carried out, and we observed ethanol FE of 5–8%, acetate and formate of 2–5%, and a trace amount of propanol. Considering the FE of acetate and ethanol plus ethylene, the GDE prepared with 20 wt.% PVP microgels produced over 70% C_2+_ products. However, the main CO_2_RR products of the microgels‐optimized GDE were gas phase with the total FE of gaseous products (H_2_, CO, CH_4_, C_2_H_4_) was over 85%; therefore we focused on the analysis/report of gaseous products in this work for both alkaline and MEA cells. The overall performance of the PVP microgel‐incorporated GDEs can be seen in Figure 5e. The GDE with 20 wt.% of PVP microgels demonstrated outstanding performance as compared with the GDE without microgels, in terms of current density and FE of ethylene both in alkaline flow cell and MEA cell. Specifically, the microgels‐incorporated GDEs could cap their highest FE of ethylene at much higher current densities, leading to a significantly better partial current density of ethylene. Furthermore, the stability of the GDE with 20 wt.% PVP microgels was tested in the MEA cell for 24 h at 350 mA cm^−2^. As can be seen in Figure 5f, the full cell voltage stayed steady in a close range and the FE of ethylene was 55–58% during the test. Comparing the performance of the GDE modified with 20 wt.% PVP microgels for ethylene production also revealed the superior performance of the GDE prepared in this study, outperforming the recent studies for CO_2_RR to ethylene (Tables S2 and S3, Supporting Information). These results in the incorporation of microgels to boost the CO_2_RR performance of conventional GDEs showcase the potential promises of gels/microgels to adjust the GDE microenvironment and CO_2_ availability and can be further extended to a wide range of microgels with fine‐tuned structure and functional groups.^[^ ^39^ ^]^

## 3 Conclusion

We presented a strategy to rationally design GDEs with CO_2_‐phil pyridine‐containing microgels to enable high‐rate CO_2_ reduction to ethylene in the flow cell and MEA CO_2_ electrolyzers. The principle behind this strategy is increasing CO_2_ availability within the catalyst layer via microgels with CO_2_‐phil heterocyclic amine moieties and 3D structure, acting as CO_2_ reservoirs. In addition, the pyridine component in the microgels can assist in CO_2_ reduction as a co‐catalyst in the proximity of Cu_2_O nanocubes, acting as dual‐function components to achieve efficient ethylene production with stability at high current densities. The results showed the importance of the microgels incorporation geometry and physical structure on CO_2_ availability, mass transfer, and CO_2_RR performance, and superior results were observed for the GDE with microgels incorporated into the catalysts and with a moderate crosslinking ratio. The GDE prepared with 20 wt.% PVP microgels and optimal crosslinking ratio showed >55% FE of ethylene at 500–700 mA cm^−2^ in alkaline flow‐cell, while for the GDE prepared without the microgels, FE of ethylene was capped at 43% at 300 mA cm^−2^. It was observed that having a microgel with pyridine moieties led to a higher FE ethylene as compared to another microgel with tertiary amine (containing diethanolamine), indicating that pyridine moieties take part in CO_2_RR in addition to acting as micro‐reservoirs to enhance CO_2_ availability. The concept was further translated to a MEA cell working with humidified CO_2,_ and an over twofold increase in partial current density of ethylene compared with the GDE without microgel was achieved. Further studies can focus on more insights into the mechanistic effects of microgel addition in catalytic pathways via DFT calculations and/or in‐situ characterizations. Due to having multiple parameters involved and multiple catalytic active sites, microgels size and interaction, and distance between the active sites or how well microgels and catalysts are mixed, DFT calculations and *operando* characterizations could provide deeper insight into the effect of microgels. In addition, more attention could be paid to the synthesis of functionalized microgels and the geometry of microgels, such as particle size, and testing microgel‐modified GDEs with acidic electrolytes to mitigate undesired CO_2_ loss in the long term.

## 4 Experimental Section

### Synthesis of Microgels

The microgels were synthesized via a surfactant‐free emulsion polymerization process.^[^ ^24^, ^30^ ^]^ Solution of 4 wt.% of 4‐vinylpyridine (PVP, Sigma) or 2‐N,N′‐(diethylamino)ethyl methacrylate (DEAEMA, Sigma) as the monomers with heterocyclic and tertiary amine groups, respectively (Figure S1, Supporting Information), and N, N′‐methylene‐bisacrylamide (0.5, 1 and 2 wt.% relative to the monomer) as the cross‐linker, was prepared in a three‐neck flask, which was fitted with a N_2_ outlet/inlet, a condenser and a mechanical stirrer (200 rms). The solution was degassed for 1 h at 40 °C in an oil bath, and then the temperature was increased to 75 °C. After that, the initiator (2,2′‐azobis (2‐methyl propionamidine dihydrochloride)), dissolved in 3 ml DI water and degassed by N_2_ for 10 min at room temperature, was injected in the three‐neck flask under a mechanical stirrer. The solution turned cloudy within 15 min of initiator injection due to the polymerization and formation of microgel suspension, and it was left overnight under continuous stirring under nitrogen protection to complete polymerization. The obtained microgel suspension was purified against deionized water by membrane dialysis (MWCO: 12–14 kDa, Sigma) for 3 days to ensure all unreacted compounds were removed. The microgels were dried in a freeze dryer to keep their integrity and were easily grounded to fine powders.

### Catalyst Synthesis

Cu_2_O nanocubes were synthesized by a typical liquid‐phase reduction method.^[^ ^20b^ ^]^ In brief, 7 mL 0.1 M CuCl_2_ ·2H_2_O was added to 280 mL of deionized water. After 5 min of stirring, 21 ml of 0.2 m NaOH solution was added to the solution, followed by a drop‐wise addition of 14 ml of 0.1 m l‐ascorbic acid. The solution was stirred vigorously for 1 h, and yellow Cu_2_O nanocubes were precipitated by centrifugation, followed by washing three times with water and ethanol.

### Fabrication of Gas‐Diffusion Electrode (GDE)

To prepare the catalyst ink, 12 mg of Cu_2_O nanocubes were dispersed in 1 ml isopropanol. Then, the dried and grounded microgels (10–30 wt.% relative to the Cu_2_O powder) were dispersed in 2 mL isopropanol. After sonication for 2 h, the catalyst and microgels dispersions were mixed, and 100 µL of Nafion solution (5 wt.%) was added to it, followed by 1 h of sonication to prepare the catalyst ink for disposition. The deposition was done via an airbrush gun (Infinity CR Plus 0.4 mm, Harder & Steenbeck) using N_2_ as the carrier gas at 0.6 bar. After drying, a layer of carbon black (Vulcan XC 72, Fuel Cell Store, in isopropanol and Nafion) was sprayed on the surface of the catalyst layer. The GDEs without a carbon top layer showed excessive voltage at even low applied currents, indicating low electrical conductivity, consistent with the literature,^[^ ^9^ ^]^ therefore for all the GDEs with and without microgels, the carbon layer was introduced as the current collector. It was also noticed that having a uniform layer of carbon with ≈ 5–7 µm thickness was sufficient for electron transfer to the catalyst layer. The GDE with microgels as a separate layer was fabricated by spraying the solution of microgels in Nafion and isopropanol on the PTFE substrate and then spraying the catalyst layer on top of it. The ink was sprayed from a 10 mm distance on a commercial PTFE membrane (Figure S2, Supporting Information) (Sartorius, 0.45 µm pore size) to achieve a catalyst loading of ≈2 mg cm^−2^.

### Electrochemical Reduction of CO_2_

CO_2_RR experiments were conducted in a gas‐fed flow cell electrolyzer (ElectroCell A/S, Denmark) (Figure S3, Supporting Information). The catholyte and anolyte (1.0 m KOH) were pumped through the cell using peristaltic pumps at a 10 ml min^−1^ flow rate. A mass flow controller was used to adjust the CO_2_ (99.9%, Coregas, Australia) flow rate (30 ml min^−1^) (Bronkhorst, Netherlands, ±1% resolution). The electrochemical measurement was controlled by a Biological potentiostat. The fabricated GDEs were used as the cathode (working electrode) with IrO_2_ as the anode, and they were separated by an anion exchange membrane (Fumasep FAA‐3‐PK‐130). An Ag/AgCl was used as a reference electrode fitted in the inlet of catholyte (Figure S3, Supporting Information) to set the potential, and the potentials were converted to reversible hydrogen electrode (RHE) scale via E (V vs. RHE) = E (V vs. Ag/AgCl) + $E_{\mathrm{Ag}/\mathrm{AgCl}}^{o}$ + 0.0591 pH with *iR* correction. $E_{\mathrm{Ag}/\mathrm{AgCl}}^{o}$ is 0.209 for the Ag/AgCl reference electrode filled with 3M NaCl solution. GDE area of 1 cm × 1 cm was exposed to the electrolyte as the active area for electrolysis. GDEs were conditioned for 1 h at −1 V vs. RHE before electrochemical tests. The dual‐layer capacitance (C_dl_) was estimated via CVs over a 0.1 V window near the open‐circuit voltage at scan rates from 20 to 100 mV s^−1^, followed by $C_{dl}=J/\left(\frac{dV}{dt}\right)$, where J is the current density in the center of 0.1 V window, and $\frac{\mathrm{dV}}{\mathrm{dt}}$ is the CV scan rate. CO_2_RR in MEA electrolyzer was done in a cell with a titanium body (Figure S5, Supporting Information), and an anion exchange membrane (Sustainion X37‐50) was used to separate cathode GDE and IrO_2_ anode GDE. Humidified CO_2_ was fed to the cathode side, and 0.1 KHCO_3_ was circulated in the anode part.

A Shimadzu GC‐2014 gas chromatograph with a ShinCarbon packed column (ST 80/100, 2 mm ID, 1/8 OD Silco, Restek) and a thermal conductivity detector (TCD) and a flame ionization detector (FID) was used to analyze the composition of gaseous products. Hydrogen (H_2_, 99.999%) and argon (Ar, 99.999%) were used as the carrier gases for the FID and the TCD, respectively. Air was used as the balance gas for the FID. The FE of gaseous products was determined via *FE_i_* = $\;\frac{e_{i}\times F\times P\times V\times X_{i}}{J\times R\times T}\times 100$, where e_i_ is the electron transfer required (in mole) to generate one mole of a gas product, X_i_ represents the product concentration in the reactor gas outlet measured with the mass spectrometer, V is the outlet gas volumetric flow rate (ml min^−1^), P is the atmospheric pressure (101.3 kPa), and J is the current (mA) (from the potentiostat). Liquid products were measured with high‐performance liquid chromatography (HPLC) (Shimadzu, Hi‐Plex H, 7.7 × 300 mm, 8 µm column, SPD‐20A/20AV UV–vis detector). The FE of liquid products was calculated using $FE_{i}=\frac{e_{i}\times F\times n}{Q}$, where e_i_ is the electron transfer for the production of liquid production from CO_2_, F is Faraday's constant (96485 C mol^−1^), and n is the moles of the produced liquid product measured via HPLC. Q represents the total charge during the experiment.

### Characterizations

A Hitachi HF5000 (accelerating voltage of 200 kV) equipped with EDAX analysis was used to acquire high‐resolution transmission electron microscopy (HRTEM) images. A JOEL‐7100F was used to achieve field emission scanning microscopy (FESEM) images. X‐ray diffraction (XRD) was obtained on a Rigaku SmartLab (Cu Kα (λ = 1.5405 Å) radiation source). X‐ray photoelectron spectroscopy (XPS) was obtained on a Kratos Axis ULTRA XPS with a 165 mm hemispherical electron energy analyzer and a monochromatic Al Kα (1486.6 eV) radiation source at 15 kV (10 mA). XPS data were analyzed by CASA software (calibrated to the C 1s signal at 284.8 eV). The hydrodynamic diameters of microgels were measured by Electrophoretic Light Scattering (Malvern, Nano‐ZS). CO_2_ adsorption–desorption isotherm was obtained by TriStar Micromeritics. CO_2_ temperature‐programmed desorption was done in the BELCAT Catalyst Characterization Analyzer (Japan), and the contact angle was captured by a Dataphysics instrument (TBU 100EC). FIB‐SEM was done using Hitachi NX5000 (Japan) and 3D reconstruction was obtained by Amira software.

## Conflict of Interest

The authors declare no conflict of interest.

## Supporting information

Supporting Information

## Data Availability

The data that support the findings of this study are available from the corresponding author upon reasonable request.
