# Supplementary material for: Rational Designing Microenvironment of Gas‐Diffusion Electrodes via Microgel‐Augmented CO2 Availability for High‐Rate and Selective CO2 Electroreduction to Ethylene
Source: Adv Sci (Weinh). 2024 Aug 29;11(40):2402964. doi: 10.1002/advs.202402964 (PMC11515925; doi:10.1002/advs.202402964)
Supplement: Supplementary file 1 — Supporting Information [file ADVS-11-2402964-s001.docx]

**Supporting Information**

**Rational designing microenvironment of gas-diffusion electrodes via microgel-augmented CO_2_ availability for high-rate and selective CO_2_ electroreduction to ethylene**

*Hesamoddin Rabiee *^a,b^, Mengran Li ^c^, Penghui Yan ^a^, Yuming Wu ^d^, Xueqin Zhang ^e^, Fatereh Dorosti ^a^, Xi Zhang ^a^, Beibei Ma ^a^, Shihu Hu ^e^, Hao Wang ^b^, Zhonghua Zhu *^a^, Lei Ge *^b,f^*

^a^ School of Chemical Engineering, The University of Queensland, Brisbane, QLD 4072, Australia. E-mail: z.zhu@uq.edu.au, h.rabiee@uq.edu.au

^b^ Centre for Future Materials, University of Southern Queensland, Springfield, QLD 4300, Australia. E-mail: lei.ge@usq.edu.au, hesamoddin.rabiee@unisq.edu.au

^c^ Department of Chemical Engineering, The University of Melbourne, VIC 3052, Australia

^d^ School of Engineering, Macquarie University, Sydney, NSW 2109, Australia

^e^ Australian Centre for Water and Environmental Biotechnology (ACWEB), The University of Queensland, St. Lucia, QLD 4072, Australia

^f^ School of Engineering, University of Southern Queensland, Springfield, QLD 4300, Australia


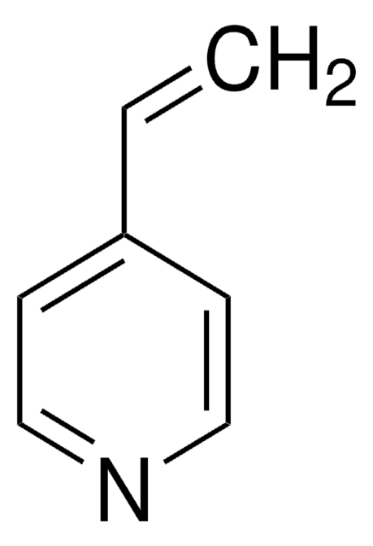

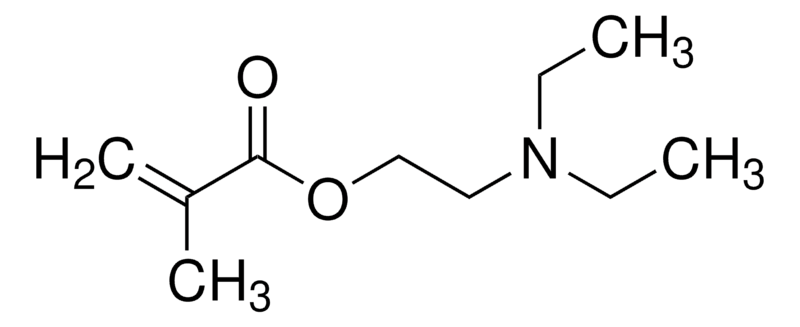


**a**

**b**

***Fig. S1.*** *Chemical structure of* ***a)*** *4-Vinylpyridine (PVP) and* ***b)*** *2-(Diethylamino)ethyl methacrylate (DEAEMA) monomers with tertiary amine groups used to synthesize microgels in this study.*


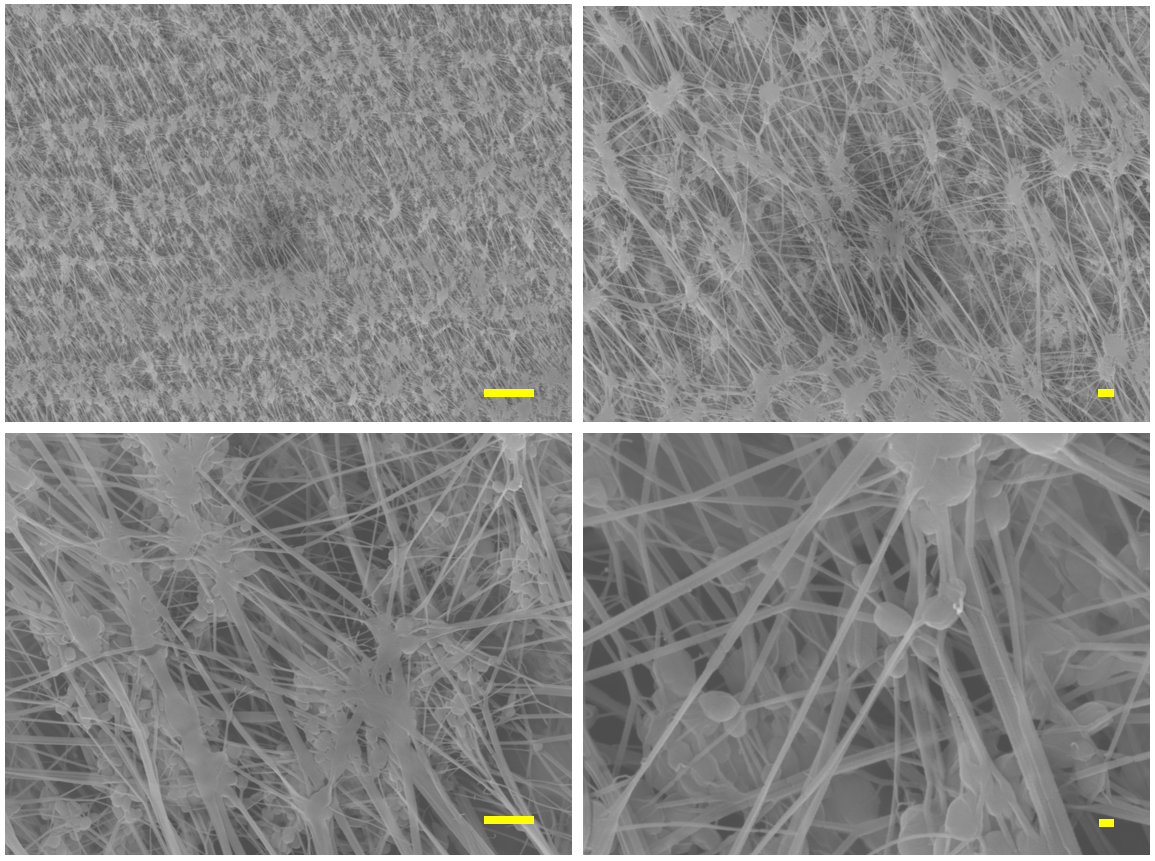


***Fig. S2.*** *SEM Images of PTFE substrate used in this study to facilitate GDEs. (scale bars: 10 µm for a, 1 µm for b and c, 100 nm for d)*


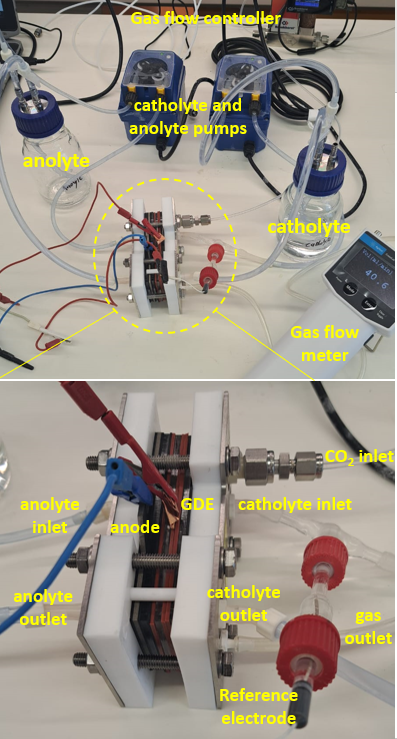


***Fig. S3.*** *Images of the flow cell CO_2_ electrolyzer with gas/liquids inlet/outlet.*


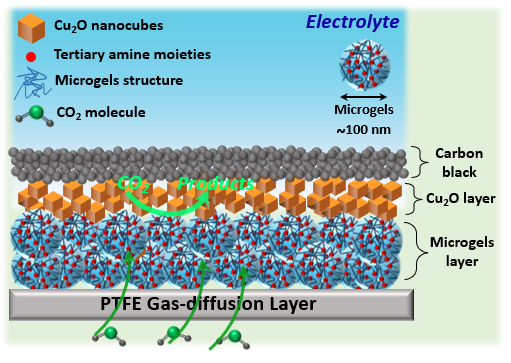


***Fig. S4.*** *Schematic of a GDE with microgels layer under the catalyst (Cu_2_O) layer, and a carbon black top-layer on PTFE substrate*


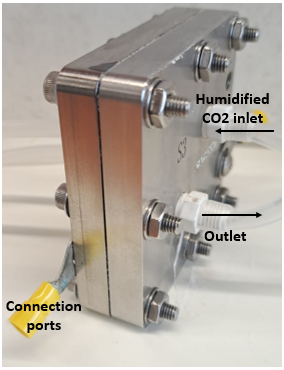


***Fig. S5.*** *Image of the MEA cell used for CO_2_RR. The cell is made of Titanium to provide conductivity. Humidified CO2 (after passing through DI water) is fed to the cathode side. 0.1 KHCO_3_ is circulated in the anode side.*


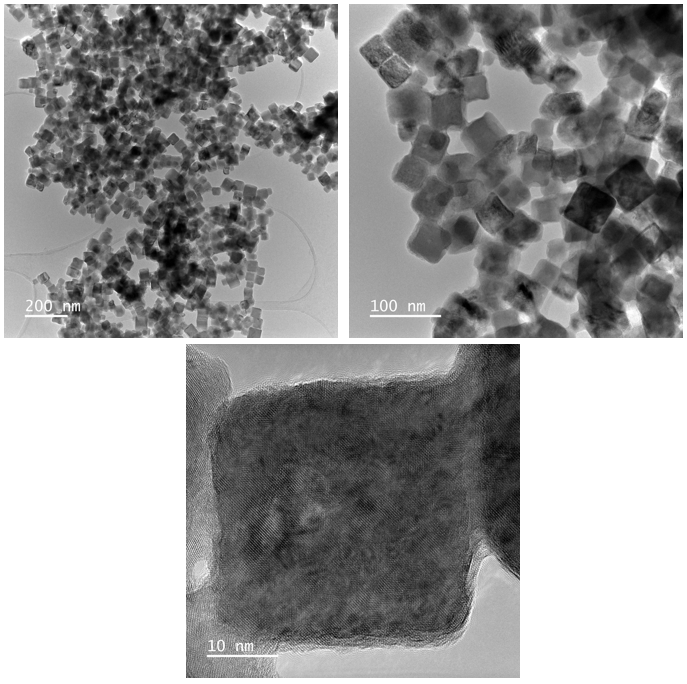


***Fig. S6.*** *HRTEM images of Cu_2_O nanocubes*


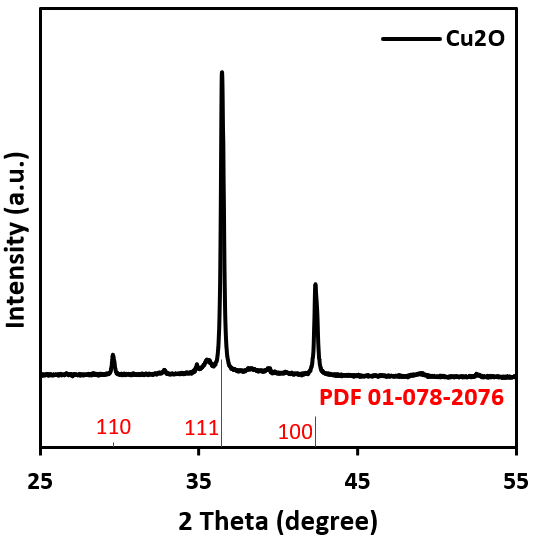


***Fig. S7.*** *XRD pattern of Cu_2_O catalyst used to fabricate GDEs.*


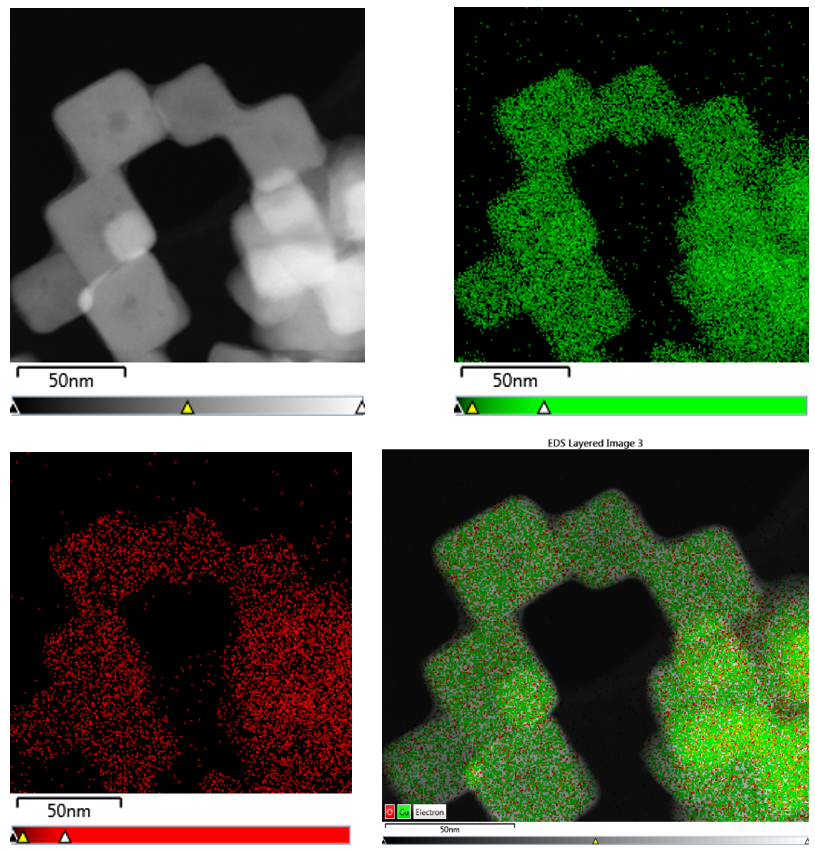


***Fig. S8.*** *a) HAADF-STEM and corresponding elemental mapping images of Cu_2_O nanocubes for* ***b)*** *Cu atom,* ***c)*** *O atom,* ***d)*** *overlap.*


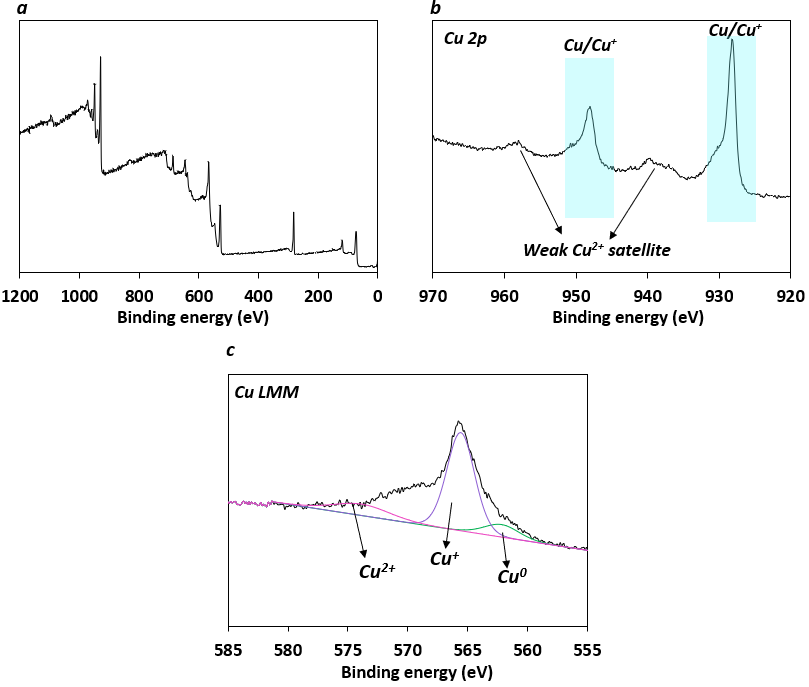


***Fig. S9.*** ***a)*** *XPS spectra of copper oxide nanocubes and high-resolution XPS spectra of* ***b)*** *Cu 2p, and* ***c)*** *Cu LMM.*


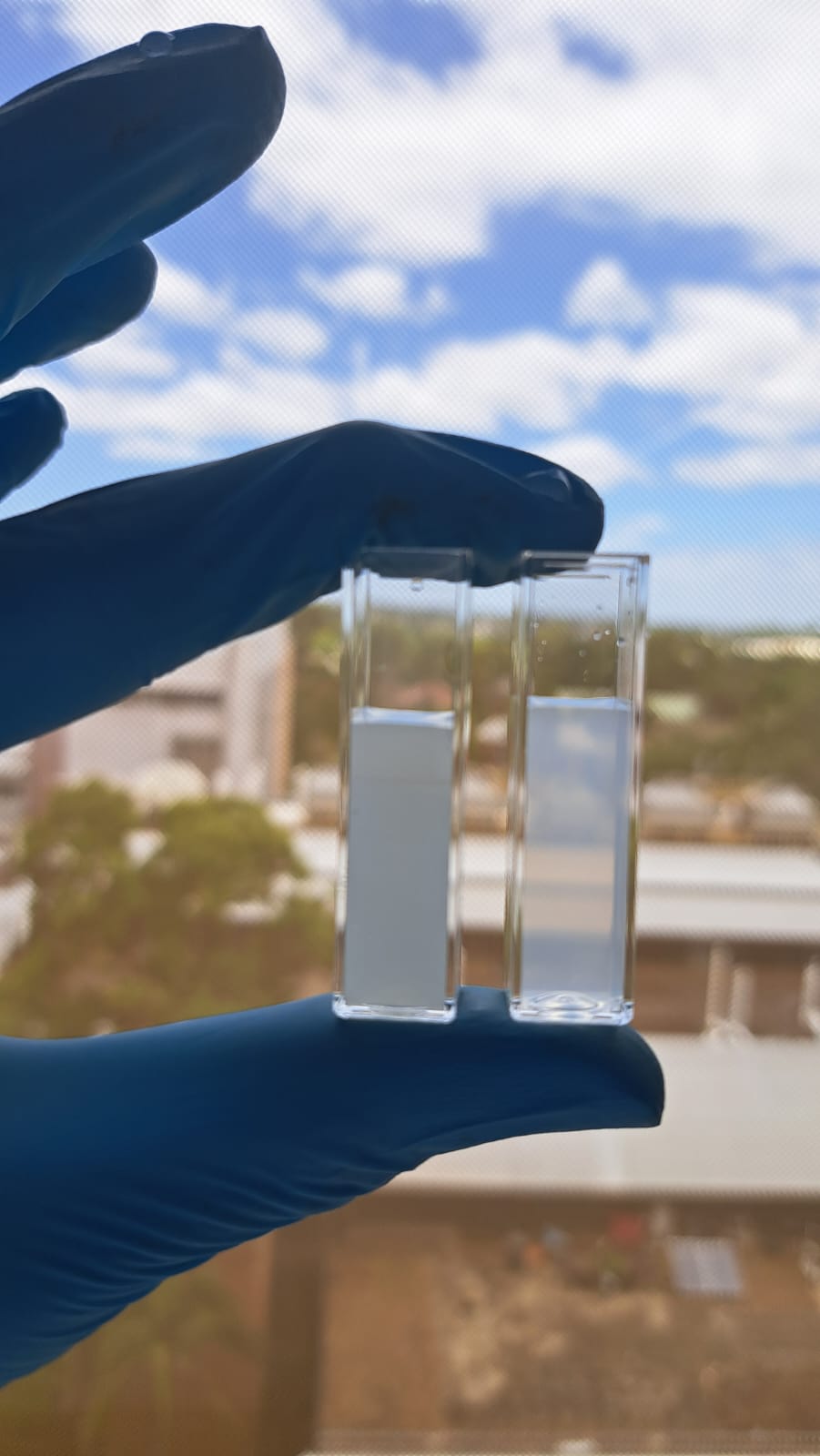


***Fig. S10.*** *Images of diluted solution of PVP microgels after saturation with N_2_ (left) and CO_2_ (right). The solution becomes transparent (right side) with CO_2_ indicating that microgels swell and enter water in their structure.*


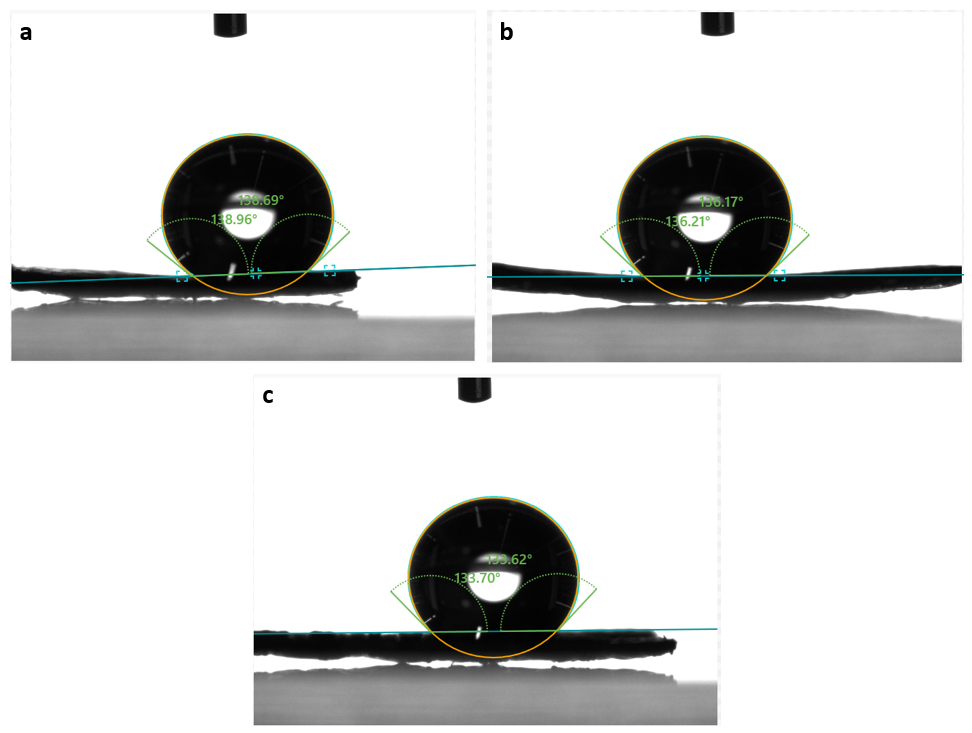


***Fig. S11.*** *Contact angle of the GDE* ***a)*** *without microgels,* ***b)*** *with 20wt% PVP microgels (before CO_2_RR tests), c) with 20wt% PVP microgels (after CO_2_RR tests)*


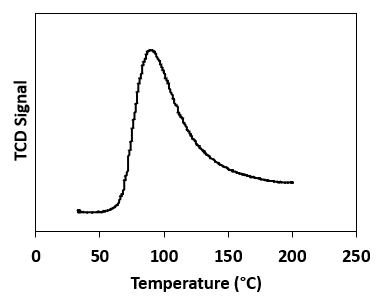


***Fig. S12.*** *CO_2_ temperature-programmed desorption (TPD) profile of PVP microgels. To do CO_2_ TPD, 200mg of the sample was put in a quartz tube and was heated to 150 C in Ar (20 ml/min) to remove any water/moisture and then cooled down to 25 C. The sample was then purged with CO_2_ at flow rate of 20 ml/min for 20 min followed by purging with Ar for 60 min to remove any CO_2_ on the surface. The sample was then heated to 200 and desorption of CO_2_ was detected by a thermal conductivity detector (TCD).*


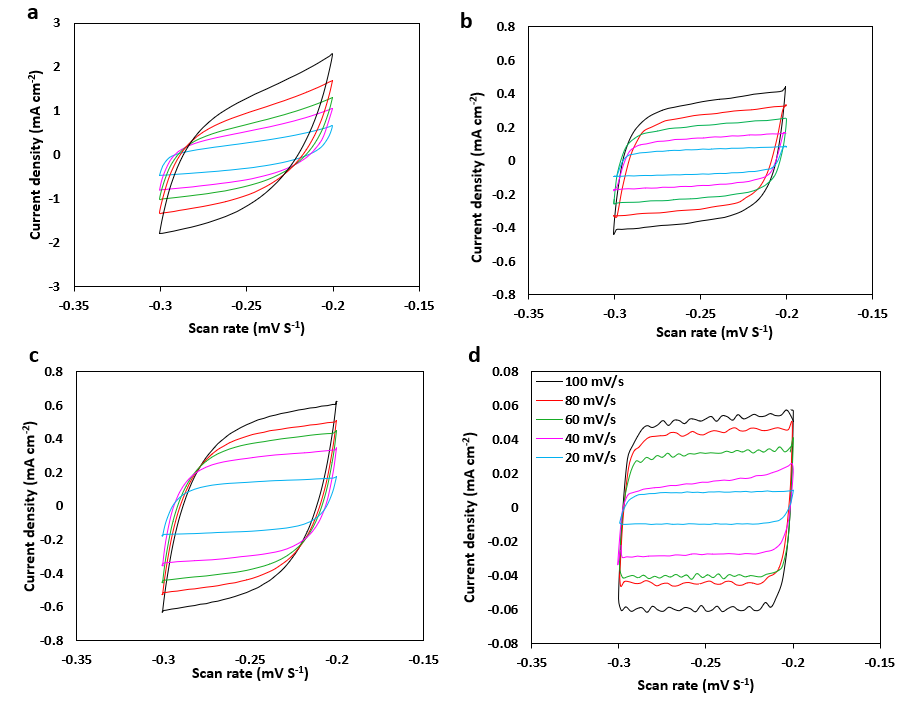


***Fig. S13.*** *Cyclic voltammetry curves for* ***a)*** *GDE without microgels, and GDE with* ***b)*** *10wt%* ***c)*** *20wt%, and* ***d)*** *30wt% PVP microgels.*

*
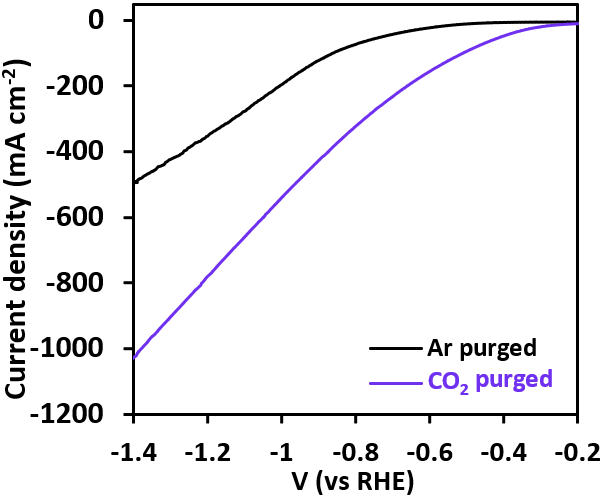
*

***Fig. S14.*** *Linear sweep voltammetry of GDE with 20wt% PVP microgels with purging CO_2_ and Ar gas.*


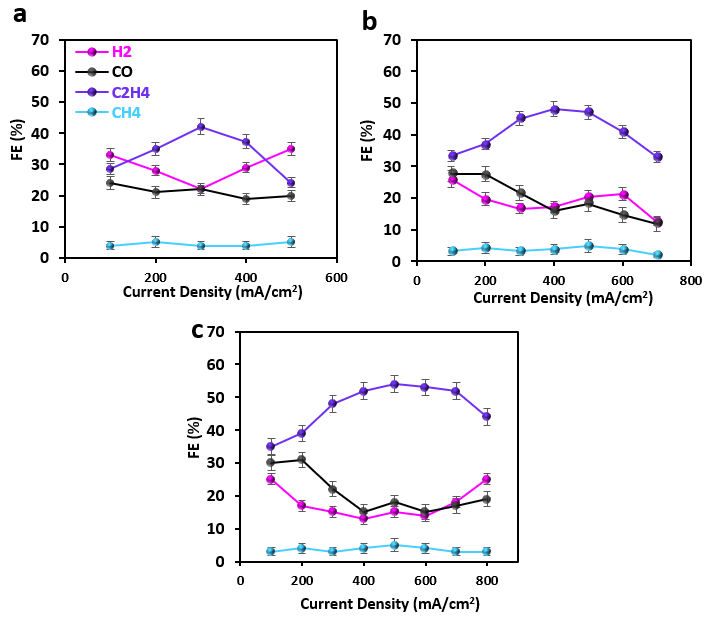


***Fig. S15.*** *CO_2_RR performance of GDEs* ***a)*** *without microgels and with* ***b)****10wt% and* ***c)*** *20wt% microgels tested in the alkaline flow cell.*


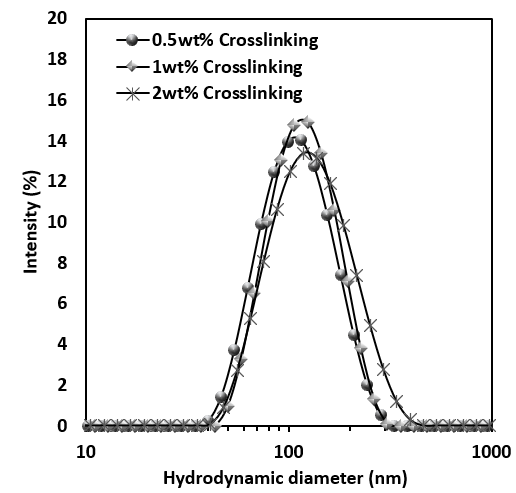


***Fig. S16.*** *Hydrodynamic diameter of PVP microgels at different crosslinking ratios.*


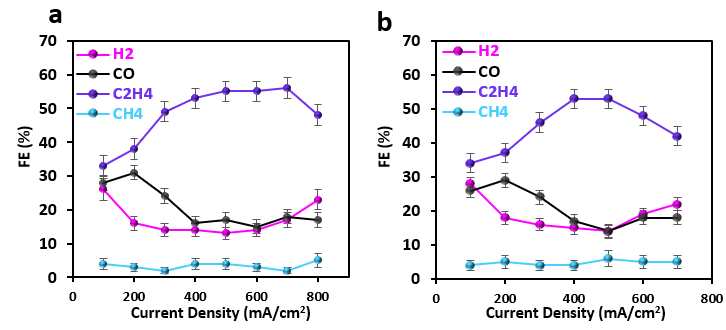


***Fig. S17.*** *CO_2_RR performance of GDEs modified with PVP microgels* ***a)*** *1wt% crosslinking* ***b)*** *2wt% crosslinking in alkaline flow cell.*


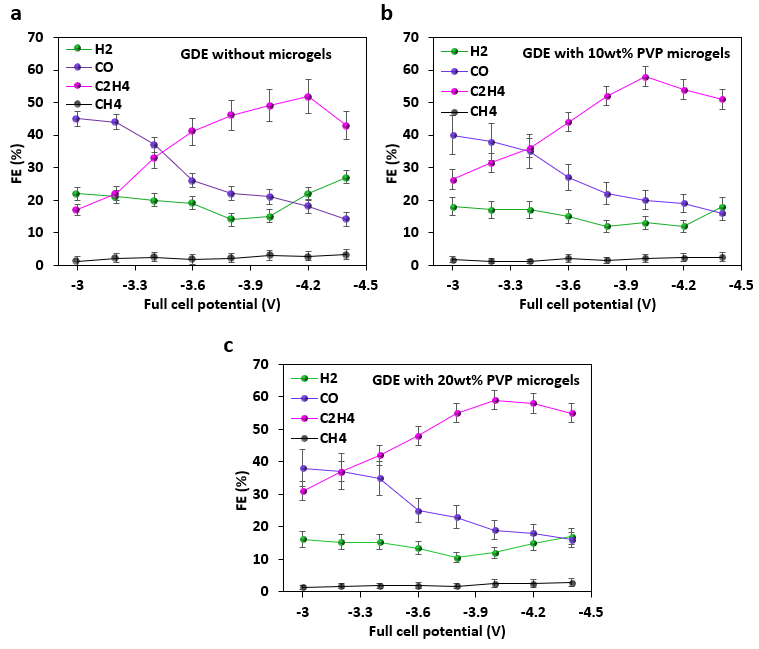


***Fig. S18.*** *CO_2_RR performance of GDEs* ***a)*** *without microgels and with* ***b)****10wt% and* ***c)*** *20wt% microgels tested in the MEA cell.*

***Table S1.*** *Diffusion layer thickness of different GDEs and various CO_2_ flow rates.*

| Samples | Gas flow rate (sccm) | R_d_ (Ω cm^2^) | D_0_ (cm^2^s^-1^) | C_d_ (mF) | δ (µm) |
| --- | --- | --- | --- | --- | --- |
| GDE without microgels | 30 | 9.164 | 0.1578 | 0.544 | 15.3±0.8 |
| GDE with 10wt% PVP microgels | 30 | 5.927 | 0.1578 | 0.521 | 12.1±0.2 |
| GDE with 20wt% PVP microgels | 30 | 2.303 | 0.1578 | 0.531 | 7.3±0.3 |
| GDE with 20wt% PVP microgels | 40 | 2.124 | 0.1578 | 0.496 | 6.9±0.4 |
| GDE with 20wt% PVP microgels | 50 | 1.944 | 0.1578 | 0.502 | 6.3 ±0.3 |
| GDE with 20wt% PVP microgels | 60 | 1.732 | 0.1578 | 0.515 | 5.9 ±0.2 |

***Table S2.*** *Performance comparison of Cu-based systems for ethylene production in flow cell*

| *Electrolyte* | *Catalyst* | *FE of ethylene (%)* | *Partial current density of ethylene (mA cm^-2^)* | *Ref.* | |
| --- | --- | --- | --- | --- | --- |
| 1 M KOH | **Cu_2_O nanocubes with PVP microgels** | **56** | **392** | **This work** | |
| 1 M KOH | Cubic Cu nanoparticles | 57 | 171 | ^[1]^ | |
| 1 M KOH | polyamine-incorporated Copper | 72 | 325 | ^[2]^ | |
| 1 M KOH | Copper Iodide | 31 | 276 | ^[3]^ | |
| 1 M KOH | Cu-S motif dispersed in framework of HKUST-1 | 57 | 228 | ^[4]^ | |
| 1 M KOH | Highly Porous Copper | 39 | 252 | ^[5]^ | |
| 1 M KOH | Dealloyed Cu-Al | 80 | 320 | ^[6]^ | |
| 1 M KOH | Copper nanocubes | 52 | 87 | ^[7]^ | |
| 1 M KOH | Cu-PzH (Pz=pyrazole) | 60 | 348 | ^[8]^ | |
| 1 M KOH | Bimetallic Cu-Pd | 47 | 169 | ^[9]^ | |
| 1 M KOH | Copper−Silver Alloys | 60 | 180 | ^[10]^ | |
| 1 M KOH | Ionomer-Modified Oxide-Derived Cu Nanosheets | 60 | 480 | ^[11]^ | |
| 1 M KOH | Cu sputtered on PTFE, MOF-modified GDE | 49.1 | 491 | ^[12]^ | |
| 3 MKOH | | Copper sputtered on PTFE | 50 | 225 | ^[13]^ |
|  |  |  |  |  | |

***Table S3.*** *Performance comparison of Cu-based systems for ethylene production in MEA cell*

| *Anolyte* | *Catalyst* | *FE of ethylene (%)* | *Partial current density of ethylene (mA cm^-2^)* | | *Ref.* |
| --- | --- | --- | --- | --- | --- |
| 0.1 M KHCO_3_ | **Cu_2_O nanocubes with PVP microgels** | **58** | **204** | **This work** | |
| 0.1 M KHCO_3_ | Molecule-hybrid Cu | 64 | 77 | | ^[14]^ |
| 0.1 M KHCO_3_ | Cu embedded by KOH | 55 | 153 | | ^[15]^ |
| 0.1 M KHCO_3_ | Molecular-doped Cu-Ag | 56 | 183 | | ^[16]^ |
| 0.1 M KHCO_3_ | Copper-silica | 65 | 215 | | ^[17]^ |
| 0.1 M KHCO_3_ | Sputtered Cu | 50 | 90 | | ^[18]^ |
| 0.1 M KHCO_3_ | Facet controlled Cu | 55 | 66 | | ^[19]^ |
| 0.5 M KHCO_3_ | Copper-oxide nanoplate array | 80 | 160 | | ^[20]^ |
| 0.1−2.0 M KOH | Porous Dendritic Copper Oxide | 41 | 62 | | ^[21]^ |
| 0.1 M KHCO_3_ | Copper Nanocubes | 40 | 80 | | ^[22]^ |
|  |  |  |  | |  |
|  |  |  |  | |  |
| 0.1 M KHCO_3_ | Cu sputtered on PTFE, MOF-modified GDE | 49.5 | 220 | | ^[12]^ |
|  |  |  |  | |  |

**References**

[1] G. L. De Gregorio, T. Burdyny, A. Loiudice, P. Iyengar, W. A. Smith, R. Buonsanti, *Acs Catal* **2020**, 10, 4854.

[2] X. Y. Chen, J. F. Chen, N. M. Alghoraibi, D. A. Henckel, R. X. Zhang, U. O. Nwabara, K. E. Madsen, P. J. A. Kenis, S. C. Zimmerman, A. A. Gewirth, *Nature Catalysis* **2021**, 4, 20.

[3] H. Li, T. Liu, P. Wei, L. Lin, D. Gao, G. Wang, X. Bao, *Angewandte Chemie* **2021**, 133, 14450.

[4] C. F. Wen, M. Zhou, P. F. Liu, Y. Liu, X. Wu, F. Mao, S. Dai, B. Xu, X. L. Wang, Z. Jiang, P. Hu, S. Yang, H. F. Wang, H. G. Yang, *Angewandte Chemie* **2022**, 61, e202111700.

[5] J. J. Lv, M. Jouny, W. Luc, W. Zhu, J. J. Zhu, F. Jiao, *Adv Mater* **2018**, 30, 1803111.

[6] M. Zhong, K. Tran, Y. Min, C. Wang, Z. Wang, C. T. Dinh, P. De Luna, Z. Yu, A. S. Rasouli, P. Brodersen, S. Sun, O. Voznyy, C. S. Tan, M. Askerka, F. Che, M. Liu, A. Seifitokaldani, Y. Pang, S. C. Lo, A. Ip, Z. Ulissi, E. H. Sargent, *Nature* **2020**, 581, 178.

[7] Y. Wang, H. Shen, K. J. T. Livi, D. Raciti, H. Zong, J. Gregg, M. Onadeko, Y. Wan, A. Watson, C. Wang, *Nano Lett* **2019**, 19, 8461.

[8] R. Wang, J. Liu, Q. Huang, L. Z. Dong, S. L. Li, Y. Q. Lan, *Angewandte Chemie* **2021**, 60, 19829.

[9] S. Ma, M. Sadakiyo, M. Heima, R. Luo, R. T. Haasch, J. I. Gold, M. Yamauchi, P. J. Kenis, *J Am Chem Soc* **2017**, 139, 47.

[10] T. T. H. Hoang, S. Verma, S. Ma, T. T. Fister, J. Timoshenko, A. I. Frenkel, P. J. A. Kenis, A. A. Gewirth, *J Am Chem Soc* **2018**, 140, 5791.

[11] Y. Zhao, X. Zu, R. Chen, X. Li, Y. Jiang, Z. Wang, S. Wang, Y. Wu, Y. Sun, Y. Xie, *J Am Chem Soc* **2022**.

[12] D. H. Nam, O. Shekhah, A. Ozden, C. McCallum, F. Li, X. Wang, Y. Lum, T. Lee, J. Li, J. Wicks, A. Johnston, D. Sinton, M. Eddaoudi, E. H. Sargent, *Adv Mater* **2022**, e2207088.

[13] M. A. Khan, S. K. Nabil, T. Al-Attas, N. G. Yasri, S. Roy, M. M. Rahman, S. Larter, P. M. Ajayan, J. Hu, M. G. Kibria, *Chem Catalysis* **2022**.

[14] F. Li, A. Thevenon, A. Rosas-Hernandez, Z. Wang, Y. Li, C. M. Gabardo, A. Ozden, C. T. Dinh, J. Li, Y. Wang, J. P. Edwards, Y. Xu, C. McCallum, L. Tao, Z. Q. Liang, M. Luo, X. Wang, H. Li, C. P. O'Brien, C. S. Tan, D. H. Nam, R. Quintero-Bermudez, T. T. Zhuang, Y. C. Li, Z. Han, R. D. Britt, D. Sinton, T. Agapie, J. C. Peters, E. H. Sargent, *Nature* **2020**, 577, 509.

[15] W. H. Lee, C. Lim, S. Y. Lee, K. H. Chae, C. H. Choi, U. Lee, B. K. Min, Y. J. Hwang, H.-S. Oh, *Nano Energy* **2021**, 84.

[16] H. Wu, J. Li, K. Qi, Y. Zhang, E. Petit, W. Wang, V. Flaud, N. Onofrio, B. Rebiere, L. Huang, C. Salameh, L. Lajaunie, P. Miele, D. Voiry, *Nat Commun* **2021**, 12, 7210.

[17] J. Li, A. Ozden, M. Wan, Y. Hu, F. Li, Y. Wang, R. R. Zamani, D. Ren, Z. Wang, Y. Xu, D. H. Nam, J. Wicks, B. Chen, X. Wang, M. Luo, M. Graetzel, F. Che, E. H. Sargent, D. Sinton, *Nat Commun* **2021**, 12, 2808.

[18] C. M. Gabardo, C. P. O’Brien, J. P. Edwards, C. McCallum, Y. Xu, C.-T. Dinh, J. Li, E. H. Sargent, D. Sinton, *Joule* **2019**, 3, 2777.

[19] G. Zhang, Z. J. Zhao, D. Cheng, H. Li, J. Yu, Q. Wang, H. Gao, J. Guo, H. Wang, G. A. Ozin, T. Wang, J. Gong, *Nat. Commun.* **2021**, 12, 5745.

[20] W. Liu, P. Zhai, A. Li, B. Wei, K. Si, Y. Wei, X. Wang, G. Zhu, Q. Chen, X. Gu, R. Zhang, W. Zhou, Y. Gong, *Nat Commun* **2022**, 13, 1877.

[21] N. H. Tran, H. P. Duong, G. Rousse, S. Zanna, M. W. Schreiber, M. Fontecave, *ACS Appl Mater Interfaces* **2022**, 14, 31933.

[22] G. O. Larrazabal, V. Okatenko, I. Chorkendorff, R. Buonsanti, B. Seger, *ACS Appl Mater Interfaces* **2022**, 14, 7779.
